# Closer look at women’s mental health

**DOI:** 10.1192/bji.2026.10099

**Published:** 2026-05

**Authors:** Hussien Elkholy

**Affiliations:** Psychiatry, Department of Clinical Neuroscience, Brighton and Sussex Medical School, Brighton, UK.

**Keywords:** Women’s mental health, perinatal mental health, menopause

It is a pleasure to announce the release of this special issue, focusing on women’s mental health (WMH).

In March this year, International Women’s Day was commemorated by the webathon Women’s Mental Health in Bloom, organised by the International Association of Women’s Mental Health^1^ and leading psychiatric organisations around the world – including the Royal College of Psychiatrists.^2^ The webathon comprised activities from different regions of the world covering various topics related to WMH.

In keeping with this spirit of inclusivity, this special issue of *BJPsych International* showcases contributions from authors representing diverse regions and perspectives, covering many facets of WMH. The opening editorial, written by Prof. ELNahas,^3^ President-elect of the International Association of Women’s Mental Health, offers a thought-provoking exploration of perinatal mental health challenges and urges action to address gaps in training, education and service provision. While the editorial centres on perinatal mental health, it also draws attention to the various factors influencing women’s mental health throughout different life stages, a theme reflected in the selection of articles in this issue.

Menopause is highlighted as another pivotal topic, given the increased vulnerability to mental health problems for some women. Hendricks et al^4^ investigate the impact of hormone replacement therapy (HRT) on depression and psychological distress among women attending a menopause clinic, reporting significant improvements in mental health outcomes.

Turning to service provision, Alabdulla et al^5^ present a narrative overview of the development of women’s mental health services in Qatar, while Hartnett et al^6^ discuss recent advances in women’s mental healthcare in Ireland. Both articles offer valuable insights into service models in different regions.

The issue also includes a range of articles on more specialised topics. I hope you find this collection both enjoyable and enriching to read.
